# Using the multiphase optimization strategy to adapt cognitive processing therapy (CPT MOST): study protocol for a randomized controlled factorial experiment

**DOI:** 10.1186/s13063-023-07669-3

**Published:** 2023-10-19

**Authors:** Rebecca K. Sripada, Cassaundra L. Peterson, John J. Dziak, Inbal Nahum-Shani, Erika M. Roberge, Amber A. Martinson, Katherine Porter, Peter Grau, Diana Curtis, Sydney McElroy, Sarah Bryant, Isabel Gracy, Cosette Pryor, Heather M. Walters, Karen Austin, Carolina Ehlinger, Nina Sayer, Shannon Wiltsey-Stirman, Kathleen Chard

**Affiliations:** 1 VA Center for Clinical Management Research, VA Ann Arbor Healthcare System, Department of Psychiatry, University of Michigan, Ann Arbor, USA; 2 VA Center for Clinical Management Research, VA Ann Arbor Healthcare System, Ann Arbor, USA; 3 Institute for Health Research and Policy, University of Illinois Chicago, Chicago, USA; 4 University of Michigan Institute for Social Research, Ann Arbor, USA; 5 VA Salt Lake City Health Care System, University of Utah School of Medicine, Salt Lake City, USA; 6 VA Salt Lake City Health Care System, Salt Lake City, USA; 7 VA Ann Arbor Healthcare System, Ann Arbor, USA; 8 Cincinnati VA Medical Center, Cincinnati, USA; 9 Center for Care Delivery and Outcomes Research, Minneapolis VA Health Care System, Minneapolis, USA; 10 VA National Center for PTSD, Stanford University, Stanford, USA; 11 Cincinnati VA Medical Center, University of Cincinnati, Cincinnati, USA

**Keywords:** PTSD, Posttraumatic stress disorder, Cognitive processing therapy, CPT, Multiphase optimization study, MOST, Factorial, Evidence-based treatment, Evidence-based psychotherapy, EBP

## Abstract

**Background:**

Approximately ten percent of US military veterans suffer from posttraumatic stress disorder (PTSD). Cognitive processing therapy (CPT) is a highly effective, evidence-based, first-line treatment for PTSD that has been widely adopted by the Department of Veterans Affairs (VA). CPT consists of discrete therapeutic components delivered across 12 sessions, but most veterans (up to 70%) never reach completion, and those who discontinue therapy receive only four sessions on average. Unfortunately, veterans who drop out prematurely may never receive the most effective components of CPT. Thus, there is an urgent need to use empirical approaches to identify the most effective components of CPT so CPT can be adapted into a briefer format.

**Methods:**

The multiphase optimization strategy (MOST) is an innovative, engineering-inspired framework that uses an optimization trial to assess the performance of individual intervention components within a multicomponent intervention such as CPT. Here we use a fractional factorial optimization trial to identify and retain the most effective intervention components to form a refined, abbreviated CPT intervention package. Specifically, we used a 16-condition fractional factorial experiment with 270 veterans (*N* = 270) at three VA Medical Centers to test the effectiveness of each of the five CPT components and each two-way interaction between components. This factorial design will identify which CPT components contribute meaningfully to a reduction in PTSD symptoms, as measured by PTSD symptom reduction on the Clinician-Administered PTSD Scale for DSM-5, across 6 months of follow-up. It will also identify mediators and moderators of component effectiveness.

**Discussion:**

There is an urgent need to adapt CPT into a briefer format using empirical approaches to identify its most effective components. A brief format of CPT may reduce attrition and improve efficiency, enabling providers to treat more patients with PTSD. The refined intervention package will be evaluated in a future large-scale, fully-powered effectiveness trial. Pending demonstration of effectiveness, the refined intervention can be disseminated through the VA CPT training program.

**Trial registration:**

ClinicalTrials.gov NCT05220137. Registration date: January 21, 2022.

**Supplementary Information:**

The online version contains supplementary material available at 10.1186/s13063-023-07669-3.

## Administrative information

Note: the numbers in curly brackets in this protocol refer to SPIRIT checklist item numbers. The order of the items has been modified to group similar items (see http://www.equator-network.org/reporting-guidelines/spirit-2013-statement-defining-standard-protocol-items-for-clinical-trials/).

**Table:** 

Title {1}	Using the multiphase optimization strategy to adapt cognitive processing therapy (CPT MOST): study protocol for a randomized controlled factorial experiment
Trial registration {2a and 2b}.	ClinicalTrials.gov Identifier: NCT05220137
Protocol version {3}	May 25, 2023; Version 1.0
Funding {4}	This research is funded by the VA Health Services Research and Development Service HSR&D I01 HX003487-01, IIR 21–088 (PI: Sripada).
Author details {5a}	Rebecca K. Sripada: VA Center for Clinical Management Research, VA Ann Arbor Healthcare System; University of Michigan Department of Psychiatry Cassaundra L. Peterson: VA Center for Clinical Management Research, VA Ann Arbor Healthcare System John J. Dziak; Institute for Health Research and Policy, University of Illinois Chicago; Inbal Nahum-Shani; University of Michigan Institute for Social Research Erika M. Roberge; VA Salt Lake City Health Care System; University of Utah School of Medicine Amber A. Martinson; VA Salt Lake City Health Care System Katherine Porter; VA Ann Arbor Healthcare System Peter Grau; VA Center for Clinical Management Research, VA Ann Arbor Healthcare System; University of Michigan Department of Psychiatry Diana Curtis; VA Center for Clinical Management Research, VA Ann Arbor Healthcare System Sydney McElroy; Cincinnati VA Medical Center Sarah Bryant; VA Salt Lake City Health Care System Isabel Gracy; VA Center for Clinical Management Research, VA Ann Arbor Healthcare System Cosette Pryor; VA Salt Lake City Health Care System Heather M. Walters: VA Center for Clinical Management Research, VA Ann Arbor Healthcare System; Karen Austin; VA Center for Clinical Management Research, VA Ann Arbor Healthcare System Carolina Ehlinger; Cincinnati VA Medical Center; Nina Sayer; Center for Care Delivery and Outcomes Research, Minneapolis VA Health Care System Shannon Wiltsey-Stirman; VA National Center for PTSD; Stanford University Kathleen Chard; Cincinnati VA Medical Center; University of Cincinnati
Name and contact information for the trial sponsor {5b}	Investigator initiated research; Rebecca Sripada (Principal Investigator); rebecca.sripada@va.gov.
Role of sponsor {5c}	This is an investigator-initiated clinical trial. The funders played no role in the design of the study; the collection, analysis, and interpretation of data; or the writing of the manuscript.

## Introduction

### Background and rationale {6a}

#### Importance of adapting CPT into a brief format

Approximately 10% of US military veterans [1, 2] suffer from posttraumatic stress disorder (PTSD). PTSD is associated with myriad negative outcomes, including lost productivity, substance use, later-life physical disability, reduced quality of life, and increased risk of suicide [3–7]. Cognitive processing therapy (CPT) is a first-line trauma-focused evidence-based psychotherapy (EBP) for PTSD with a large effect size in efficacy studies [8]. CPT is recommended as a first-line treatment by the American Psychological Association, [9] the Department of Veterans Affairs (VA) and Department of Defense [10], and the International Society for Traumatic Stress Studies [11] and has been highly implemented within the VA healthcare system [12]. It is initiated three times more frequently than any other trauma-focused psychotherapy in the VA [13–15] and may be preferred by veterans over other types of trauma-focused psychotherapy [16].

CPT is designed to be a time-limited treatment that consists of discrete therapeutic components delivered across 12 sessions, [17] and many patients report clinically significant improvement within eight sessions [18–20]. However, among those who initiate CPT, 60–70% drop out before receiving eight sessions of treatment, [13, 15] and those who drop out receive only four sessions on average [13, 21]. Veterans drop out for multiple reasons, but the most widely cited reasons are time constraints, logistical barriers, and lack of perceived benefit [22, 23]. Unfortunately, veterans who drop out prematurely may never receive the most effective components of the 12-session CPT protocol. In recognition of the problem of attrition, the National Research Action Plan, a unified mental health research plan spanning three government agencies (National Institutes of Health, Veterans Health Administration, and Department of Defense), has prioritized work to “Enhance current PTSD evidence-based treatment delivery to be briefer, more durable, and more efficacious” [24]. Thus, a brief format of CPT is critically needed to ensure receipt of the most effective components of treatment, resulting in high-quality PTSD care.

Although modular versions of CPT have been proposed [18, 25], these protocols have not been based on empirical evidence of component effectiveness, nor are they brief enough to meet the needs of veterans in clinical practice [13, 26]. Thus, there is an urgent need to systematically optimize a brief CPT in order to reduce dropout. The 70% who drop out prematurely will likely continue to experience symptom-related distress and numerous other negative outcomes [3–7].

#### Theoretical framework

The theoretical framework for this study is a Forms and Functions-informed [27] version of Social Cognitive Theory for PTSD [28]. Social Cognitive Theory posits that individuals learn causal rules about behavior from an early age and use them as organizing principles to help understand actions and consequences, such as the “just world belief,” or the belief that “everything happens for a reason.” When a traumatic event occurs and needs to be integrated into the belief system, individuals either use previously learned rules to make sense of the event or change their beliefs to fit the event. CPT uses a variety of tools to identify and correct skewed beliefs, which in turn leads to reductions in PTSD and depression [29]. The Forms and Functions model of complex health interventions can be used to facilitate these cognitive changes more efficiently. This model posits that Core Functions are the key processes that the intervention facilitates, and Forms are the different tools or strategies that can be used to carry out the Core Functions [27]. The Core Functions in CPT are skills that lead to reductions in maladaptive trauma-related beliefs, a key mechanism of change in CPT [30]. The Forms are the different therapeutic tools used to activate, teach, or build these skills and achieve this reduction. The Forms and Functions model advocates for selecting the Form(s) that best achieve the desired function. To identify the best Forms for this purpose, we will use a highly efficient experimental design, as described below.

#### The multiphase optimization strategy (MOST) framework can be used to guide improvements to behavioral interventions through highly efficient experimentation

The MOST uses ideas from engineering and manufacturing, such as sequential improvement, the use of factorial experiments to screen for important factors influencing product quality, and the use of a pre-specified optimization criterion to define good performance [31]. MOST follows a resource management principle, which dictates that resources be carefully managed to provide maximal information from a given design [31]. This principle guides the optimization phase of MOST, in which intervention components are tested to determine whether they should be included in the intervention package. Factorial designs can be used in the optimization phase of MOST to efficiently address scientific questions about the selection of multiple intervention components. In a full factorial design, all potential combinations of intervention components are tested, such that the number of experimental conditions increases to the extent that more components are tested. In a fractional factorial design, only a carefully selected fraction of the conditions is tested [32]. A fractional factorial experiment is consistent with the resource management principle because it includes fewer experimental conditions while still testing component effects that are considered to be most important (e.g., main effects and two-way interactions) [32]. Following the factorial experiment, an intervention package can be assembled and evaluated in a follow-up evaluation RCT.

### Objectives {7}

The long-term goal of this line of research is to adapt, test, and implement brief, evidence-based treatment for veterans with PTSD. The overall objective is to optimize a brief version of CPT and identify mediators and moderators that will ultimately allow a more tailored brief approach. Identifying the most effective intervention components and delivering only those components will make CPT deliverable in a shorter timeframe, thus improving efficiency, reducing drop-out, [23] and ensuring that veterans receive the most beneficial components of treatment, which will likely improve their quality of life [3]. The feasibility of our objective is supported by our team’s experience designing optimization trials, [33, 34] delineating the treatment adaptation process [35], adapting CPT to improve outcomes [36], and implementing CPT across the VA system [12]. Identifying moderators of component effectiveness will enable us to further tailor the delivery of specific components in future work. To accomplish our objective, guided by the MOST framework, we designed a fractional factorial experiment to test the effectiveness of each component and each two-way interaction between components. The trial will enroll 270 veterans at three VA Medical Centers (VAMCs) and all components will be delivered in-person or by telehealth. Our specific aims are:

- Specific Aim 1: Using a highly efficient experimental design, identify which of five CPT components contribute meaningfully to reduction in PTSD symptoms. We will test the effectiveness of each component and each two-way interaction between components, as measured by PTSD symptom reduction on the Clinician-Administered PTSD Scale for DSM-5 (CAPS-5) across 6 months of follow-up.
- Specific Aim 2: Identify mediators of component effectiveness. We hypothesize that effects will be mediated by engagement/adherence and change in posttraumatic cognitions.
- Exploratory Aim 1: Identify moderators of component effectiveness**.** We will test whether specific components and combinations of components are differentially effective by participant characteristics, including sex, age, and initial PTSD symptom severity.

Upon completion of these aims, our expected outcome is a refined, abbreviated version of CPT that consists of the most effective elements of the intervention. Pending demonstration of effectiveness, the refined intervention can be disseminated through the VA CPT training program and is likely to have a positive impact on the health and wellbeing of veterans with PTSD.

### Trial design {8}

Guided by the MOST framework, the goal of the proposed project is to empirically inform an abbreviated version of CPT via a highly efficient fractional factorial design. Veterans (*N* = 270) at three VAMCs (Ann Arbor, Cincinnati, and Salt Lake City) with clinically significant PTSD symptoms who meet minimal inclusion/exclusion criteria will be randomized. The primary outcome is PTSD severity, as measured by the Clinician-Administered PTSD Scale for DSM-5 (CAPS-5), [37] which will be administered by independent evaluators. This measure will be administered at baseline, post-treatment, 3-month, and 6-month post-randomization. Secondary outcomes include functioning, depression, patient satisfaction, and service utilization.

## Methods: participants, interventions, and outcomes

### Study setting {9}

We will recruit veterans from the PTSD Clinical Teams (PCTs) of three VAMCs: Ann Arbor VAMC, Cincinnati VAMC, and Salt Lake City VAMC. These VAMCs were selected based on CPT clinical trial experience, patient capacity, and therapist willingness to allocate time towards 4–8 sessions of therapy per participant and additional follow-up, as needed.

### Eligibility criteria {10}

Our sample is designed to be representative of veterans with PTSD who generally receive treatment within PCTs, with minimal exclusion criteria. Inclusion criteria for PTSD symptoms is a score of 33 or above on the PTSD Checklist for DSM-5 (PCL-5) [38] when anchored to an index trauma, and a subsequent diagnosis of PTSD based on the CAPS-5 [37], a structured clinical interview for PTSD. Individuals taking psychotropic medication must have at least four weeks on a stable dose. Participants must also be at least 18 years of age. Exclusion criteria include acute suicide risk requiring hospitalization or higher intensity treatment, need for detoxification, severe cognitive impairment indicated via electronic health record or in the judgment of the study staff (as evidenced by confusion, inability to follow the discussion or answer questions, or other clear and significant indicators of cognitive impairment), current psychosis or unmanaged bipolar disorder, previous lifetime receipt of CPT, and current or past-year engagement in any trauma-focused psychotherapy (e.g., Prolonged Exposure, Written Exposure Therapy, or Eye Movement Desensitization and Reprocessing).

### Who will take informed consent? {26a}

As part of the informed consent process, research study staff will explain to patients the nature of study activities, that participation is voluntary, and that individuals may withdraw at any time without repercussion. Participants will be given a copy of the consent form. The informed consent process will be conducted by study staff using the IRB-approved and stamped form following eligibility screening. DocuSign will be used to obtain written informed consent for all patients. If a patient is unable to use DocuSign for any reason, we will work with participants to obtain a hard copy, signed informed consent form.

### Additional consent provisions for collection and use of participant data and biological specimens {26b}

Additional consents are not collected.

## Interventions

### Explanation for the choice of comparators {6b}

The analysis of a fractional factorial design is based on comparing means of combinations of experimental conditions in order to estimate the main effects and interactions of treatment components (see the “Intervention description {11a}” section).

### Intervention description {11a}

All participants receive between four and eight CPT sessions. These sessions mirror the standard CPT protocol. The first two sessions are consistent across conditions and provide core components of CPT.

#### CPT core components/functions

The core functions of CPT are cognitive skills that lead to reductions in negative posttraumatic cognitions, and specifically assimilated and over-accommodated beliefs [30]. The core components delivered in sessions 1 and 2 lay the groundwork for achieving these core functions. In the first session, the therapist provides a rationale for treatment via CPT and teaches the veteran how to construct an Impact Statement, which describes the veteran’s beliefs about the cause of the index trauma. During the second session, the therapist uses the Impact Statement to identify the stuck points that become the focus of treatment and teaches the veteran how to distinguish between events, thoughts, and feelings using the A-B-C worksheet. During the first two sessions and in all subsequent sessions, the therapist uses Socratic questioning. Socratic questioning is an open-ended style of questioning in which the therapist adopts a stance of respectful curiosity in order to help the veteran come to his or her own realizations about the inaccuracy of their assimilated and over-accommodated beliefs (“stuck points”). Therapist skill in Socratic questioning is highly predictive of greater symptom improvement [30]. Each participant then receives between two and six additional weekly sessions, as described below.

#### Tested components

All five remaining CPT components (“Forms” in the Forms and Functions model) will be empirically tested in the fractional factorial design (see Table 1). Each component will be taught in a single session. For example, a participant randomized to Condition 4 will receive the two initial core sessions, the Modified A-B-C session, the Challenging Questions session, and Module Choice session, and the final session (see Table 1). As in standard CPT, each session will begin with a review of homework from the previous session and then provide the listed component. The tested components are (1) Modified A-B-C, (2) Challenging Questions, (3) Patterns of Problematic Thinking, (4) Challenging Beliefs, and (5) Veteran’s choice of Module (Safety/Trust/Power and Control/Esteem/Intimacy). The Modified A-B-C session reinforces the connections between events, thoughts, and feelings (from the original A-B-C session) and incorporates preliminary cognitive restructuring to help challenge stuck points. The Challenging Questions session teaches the veteran to question and confront maladaptive self-statements and stuck points. The Patterns of Problematic Thinking session teaches the veteran to identify counterproductive thinking patterns. The Challenging Beliefs session teaches the veteran to generate alternative thoughts based on the outcome of cognitive restructuring. The Module Choice session encourages cognitive flexibility regarding specific themes that are commonly affected by traumatic events (Safety Issues, Trust Issues, Power and Control Issues, Esteem Issues, and Intimacy Issues). For conditions that include the Module Choice component, the therapist will present the Module options to the veteran, and the veteran will select one Module to work on. The final session consists of homework review, review of treatment progress, introduction to the CPT coach mobile app, and encouragement of continued practice.

#### Fractional factorial design

**Table 1:** Fractional factorial design

Condition	Core Session 1 (Brief Psychoeducation, Impact Statement)	Core Session 2 (A-B-C)	Modified A-B-C	Challenging Questions	Problematic Patterns	Challenging Beliefs	Module Choice	Final Session
1	On	On	Off	Off	Off	Off	On	On
2	On	On	On	Off	Off	Off	Off	On
3	On	On	Off	On	Off	Off	Off	On
4	On	On	On	On	Off	Off	On	On
5	On	On	Off	Off	On	Off	Off	On
6	On	On	On	Off	On	Off	On	On
7	On	On	Off	On	On	Off	On	On
8	On	On	On	On	On	Off	Off	On
9	On	On	Off	Off	Off	On	Off	On
10	On	On	On	Off	Off	On	On	On
11	On	On	Off	On	Off	On	On	On
12	On	On	On	On	Off	On	Off	On
13	On	On	Off	Off	On	On	On	On
14	On	On	On	Off	On	On	Off	On
15	On	On	Off	On	On	On	Off	On
16	On	On	On	On	On	On	On	On

The optimization phase of the MOST framework is intended to empirically inform an intervention package that is not only effective, but also practical (i.e., feasible given real-world constraints). This phase involves conducting an optimization trial using an efficient experimental design, such as the factorial experiment, to identify which combination of components is most likely to meet the optimization criterion (e.g., to have high efficacy while including no inactive components). In the current setting, a full factorial design would involve five factors (one for each of the components under investigation), each involving two levels: On (when a component will be offered) and Off (which a component will not be offered), requiring 2^5^ = 32 experimental conditions. Such a large number of experimental conditions would be impractical to carry out in the current setting due to the logistical challenge of ensuring therapist fidelity to an overwhelming number of conditions. Fractional factorial designs make highly efficient use of experimental subjects, while involving only a subset of the experimental conditions in a standard factorial design [32]; they provide an alternative for investigators who wish to take advantage of the efficiency of factorial experiments but have the resources to implement only a limited number of experimental conditions [39]. Hence, we opted to conduct a half-fractional factorial, described in Table 1. This particular 2^5–1^ factorial, which includes only half (i.e., 16) of the experimental conditions required in a standard factorial design, is known as a Resolution V design [32, 33]; it is designed to test main effects and two-way interactions, with the assumption that higher-order interactions (i.e., three-way and higher) between components are negligible [11]. This assumption is based on the effect hierarchy principle, stating that the higher the order of the effect, the less likely it is to be important scientifically [32, 33]. In contrast, a standard evaluation RCT is unable to test any main effects of treatment components or interactions between treatment components.

It is important to note that the factorial design in Table 1 should not be considered a 16-arm trial in which each experimental condition is compared to a control condition. Analysis of a 16-arm RCT would require comparing 16 individual experimental conditions and, hence, would be grossly underpowered. Instead, each main effect and 2-way interaction will be tested by utilizing data from all 16 experimental conditions. For example, the main effect of Challenging Beliefs will be tested by comparing the mean CAPS-5 change among participants (*n* = 135) in the experimental conditions in which this component was set to ON (i.e., those in conditions 9–16 in Table 1) versus those in the experimental conditions (*n* = 135) in which this component was set to Off (i.e., those in conditions 1–8). This is similar to a two-arm comparison between half of the sample (i.e., those who were offered Challenging Beliefs) and the other half (i.e., those who were not offered Challenging Beliefs). Because the factorial design compares means based on combinations of experimental conditions, a factorial experiment can be adequately powered even with a relatively small number of experimental subjects per condition [32, 33]. Our power calculation allows for the detection of main effects and two-way interactions between components. The analysis will be intent-to-treat.

CPT will be delivered by telehealth (VA Video Connect) or in person, depending on participant preference and current VAMC practices. Study team members have previously demonstrated equivalency of CPT when delivered by telehealth versus in-person [40]. The components that are essential for achieving core functions (CPT core components) will be provided to every participant. All other CPT components will be empirically tested. We will audio-record all sessions and assess treatment fidelity by reviewing a randomly selected 50% of session recordings across sites. In total, veterans will receive between four and eight sessions, delivered weekly.

### Criteria for discontinuing or modifying allocated interventions {11b}

Participants are assigned to receive between four and eight sessions of CPT depending on the condition to which they are randomized. These sessions are intended to be delivered at a frequency of once per week, and as a result the intervention is expected to last four to eight weeks. This schedule may be modified based on therapist availability, clinic policy, or participant request. Participation is voluntary and participants may choose to discontinue therapy at any time. Participation may also be terminated if a therapist or investigator determines it is not in the participant’s best interest to continue (e.g., due to change in risk or clinical needs). Regardless of any changes to the intervention schedule, the intervention components within the assigned condition do not change.

On occasion, the allocated intervention may be modified by the addition of a “stressor session.” Should a participant experience a major psychosocial stressor during a course of treatment, therapists will ask if the participant would like to engage in a stressor session, in which the therapist provides support and helps the patient apply current CPT skills to the issue at hand. If administered, the stressor session does not count against the allocated number of sessions.

### Strategies to improve adherence to interventions {11c}

#### Therapist and evaluator training and consultation

The intervention will be delivered by existing clinic therapists with CPT “provider status,” indicating previous training and consultation in CPT. First, Co-Is Wiltsey-Stirman and Chard will provide therapists with a manualized 2-h CPT booster training, as in their previous work, [41] that includes training on each of the 16 study conditions. After the booster training, consultation groups will meet weekly. Consultation will be delivered in a structured format based on the effective standard CPT consultation model developed by study team members [42, 43]. These 1-h meetings will consist of fidelity (adherence and competence) feedback based on discussion of cases, with guidance on challenges to CPT fidelity and study fidelity (e.g., for a given condition, how to deliver the assigned components without incorporating unassigned components).

Independent evaluators will complete CAPS-5 training provided virtually by the VA National Center for PTSD and will achieve 90% or more agreement prior to conducting assessments. Assessments will be conducted by telephone or video call. Independent evaluators attend a biweekly CAPS supervision session led by Co-I Roberge.

#### Fidelity measures

Therapists will conduct each CPT session with the aid of established *Session Content Checklists* to ensure fidelity [17]. We have also developed condition-specific therapist manuals for each treatment arm, which consist of the session content checklists for each component to be provided in that condition. We will audio-record all sessions and assess treatment fidelity by *Session Audio Fidelity Monitoring*, in which we will review a randomly selected 50% of session recordings. Trained fidelity evaluators will review these sessions for fidelity (adherence and competence) using a modified version of the *CPT Fidelity Measure* that has been used in previous clinical trials by study team members [42, 43]. The CPT fidelity measure examines therapists’ adherence to specific CPT sub-components (dichotomous measure) as prescribed in each session, and their competence or skill in delivering them (7-point Likert-type scale, from 0 = not competent to 6 = outstanding competence). A mean score of all unique and essential items per session is calculated to determine adherence and competence scores. We will also assess monitor sessions for disallowed components (i.e., components that are not part of the assigned condition). Sessions will be continuously uploaded by therapists to a secure server. The study team will address incidents of inadequate fidelity in consultation calls and provide remedial training as needed.

All CAPS-5 interviews will be digitally recorded. Twenty percent of interviews will be randomly selected in an ongoing way in order to monitor the reliability of the assessment. Feedback to independent evaluators will be provided on a regular basis.

### Relevant concomitant care permitted or prohibited during the trial {11d}

Participants are asked not to enroll in another research study simultaneously without prior authorization from the study team. Participants are also asked not to engage in other trauma-focused treatments during their enrollment period unless deemed clinically appropriate or in the participant’s best interest. All therapy received during the study will be tracked.

### Provisions for post-trial care {30}

Participation lasts about 6 months. Should a participant wish to be connected to other mental health treatment after this time, the research study team will work with the participant’s local VAMC to connect them to care as deemed appropriate by their clinical providers.

Although the anticipated risk of injury is minimal, if a participant is injured as a result of taking part in this study, the VA will provide necessary medical treatment at no cost, unless the injury is due to non-compliance by a study participant with study procedures or if the research is conducted for VA under contract with an individual or non-VA institution.

Participants do not give up any legal rights or release the VA from any liability by consenting to participate in this study.

### Outcomes {12}

Veterans (*N* = 270) at three VAMCs (Ann Arbor, Cincinnati, and Salt Lake City) with clinically significant PTSD symptoms who meet minimal inclusion/exclusion criteria will be consented, enrolled, and randomized. The primary outcome is PTSD severity as measured by the Clinician-Administered PTSD Scale for DSM-5 (CAPS-5), which will be administered by independent evaluators. This measure will be administered at baseline, post-treatment, 3-month, and 6-month post-randomization. Secondary outcomes include functioning, depression, patient satisfaction, and service utilization (see Table 2).

**Table 2:** Measures

Measures	Time point
**Screening**	
PTSD symptoms (PCL-5)	Screening
Trauma history (THS)	Screening
Psychiatric comorbidities (DIAMOND)	Screening
PTSD symptoms (CAPS-5)	Screening
Suicide risk (C-SSRS)	Screening
**Primary outcome**	
PTSD symptoms (CAPS-5)	Baseline, post-tx, 3-month, 6-month
**Secondary outcomes**	
PTSD symptoms (PCL-5)	Baseline, post-tx, 3-month, 6-month
Functioning (B-IPF)	Baseline, post-tx, 3-month, 6-month
Depression (PHQ-9)	Baseline, post-tx, 3-month, 6-month
Suicide risk (C-SSRS)	Baseline, post-tx, 3-month, 6-month
Pain Catastrophizing Scale	Baseline, post-tx, 3-month, 6-month
WHO Disability Assessment Schedule (WHODAS 2.0)	Baseline, post-tx, 3-month, 6-month
Patient satisfaction (CSQ-8)	Baseline, post-tx
Service utilization	Baseline, post-tx, 3-month, 6-month
**Mechanisms and/or mediators**	
Posttraumatic cognitions (PTCI-9)	Baseline, post-tx, 3-month, 6-month
Treatment engagement	Weekly during treatment
**Moderators**	
Demographics	Baseline
Psychiatric comorbidities (DIAMOND)	Baseline
Trauma type (THS)	Screening
**Fidelity measures**	
Content checklists	Weekly
Review session audio	Weekly
CPT fidelity measure	Weekly

### Participant timeline {13}

#### Timing

##### Pre-enrollment

Screening: Screening measures are valid for 30 days from the date of completion. If a participant does not meet the criteria on one or more measures, they may be rescreened in 30 days. Alternatively, if a participant does not complete their informed consent within 30 days of their initial screening, screening measures must be completed again to verify inclusion criteria have been met.

Informed consent: Informed consent will be completed within 30 days of screening.

Baseline Symptom Interview, CAPS-5: Baseline Symptom Interviews should be completed as soon as possible after consent and within 30 days of the screening measures.

##### Enrollment

The Assessor informs the participant of eligibility at the end of the baseline symptom interview. Once a participant has been deemed eligible by the Assessor, the study team immediately notifies the local site RA. Similarly, if the participant is deemed ineligible, the Assessor informs the participant of this decision at the end of the interview and explains that no further study activities are required. If eligibility cannot be determined at completion of the symptom interview, the Assessor informs the coordinating center study team and the PI. This determination is also immediately relayed to the participant via the Assessor. Eligibility determination is not delayed more than 24 h unless authorized by the PI.

##### Post-enrollment

Therapist assignment: The local site RAs manage therapist assignment via local channels and procedures. A local therapist is assigned as soon as possible, but no later than three business days. Once assigned, the local site RA informs the coordinating study team.

Therapy scheduling: Therapy session scheduling is handled directly by the assigned therapist. The therapist will reach out to the participant as soon as possible after assignment to schedule the first session, according to local policy. If unable to contact the participant after local procedures have been exhausted, the therapist informs the local site RA. If a participant cannot be reached before randomization, the participant is considered withdrawn and will discontinue follow-up. If this occurs after randomization, the participant may withdraw from therapy but may continue follow-up if desired.

#### Intervention

##### First therapy appointment

The first therapy appointment is scheduled as quickly as possible, but with consideration to therapist schedule and participant availability.

##### Randomization

Randomization occurs after the participant attends their first therapy session. The therapist confirms attendance with the local site RA. Once notified, the local RA requests randomization from the coordinating center. Once randomization is performed, the coordinating center conveys the intervention condition assignment to the local RA. Although we aim to complete this process as quickly as possible once attendance is confirmed, it is permissible for randomization to be delayed as late as the second session, because sessions 1 and 2 are consistent across conditions.

##### Remaining sessions

The remaining number of therapy sessions ranges between two and six depending on which condition a participant is assigned to. Therapists follow local clinic policy for scheduling, cancellations, or no-shows. If a participant is considered a no-call, no-show for two consecutive appointments, they are considered dropped from the therapy. They may continue follow-ups if desired.

#### Follow-ups

Follow-up target dates are based off the date of the first therapy session and occur approximately 42 days (post-treatment), 91 days (3 months), and 183 days (6 months) after. If unable to complete assessments, the participant is considered “timed-out.” After completion of the 6-month study activities/close of window, the coordinating center notifies the local site study team of participant study completion.

### Sample size {14}

We propose to enroll 270 participants. As described above, a CPT component will be considered effective if its presence produces a statistically significant main effect or two-way interaction of Cohen’s *d* ≥ 0.25. A Cohen’s *d* of 0.25 is equivalent to a 5-point difference on the CAPS, which is recognized as the minimum threshold for therapeutic response [37]. We will consider a component to be “possibly effective” if its main effect is significant but between 0.15 and 0.25. The sample size of 270 was planned to provide 80% power to detect at least Cohen’s *d* = 0.25 standardized effect size with 0.05 two-sided tests for the outcome of PTSD symptom change. This assumes 10% lost to assessment at the primary endpoint of 6 months post-randomization, and within-person correlation of 0.7. Thus, we are adequately powered to meet the primary aim, as these anticipated effect sizes are consistent with the smallest clinically meaningful difference [37].

### Recruitment {15}

Historically, across our three recruitment sites, CPT is delivered to over 750 veterans per year. Based on enrollment data from previous CPT clinical trials at our recruitment sites, we expect that ~ 75% will meet inclusion criteria, and we conservatively estimate that 25% will be willing to participate. Thus, approximately 140 veterans per year will be eligible, and enrollment of 90 veteran participants per year is highly feasible. In prior studies conducted by study team members at these recruitment sites, 94% of enrolled participants have initiated treatment [44]. In order to enroll 270 veterans over the course of the 3-year enrollment period, we will need to enroll approximately eight veterans per month. Our strategy for enrolling participants at this pace is primarily through clinic referrals but may also be from self-referral or screening electronic health records. To ensure adequate representation of female and racial/ethnic minority veterans, we will carefully monitor enrollment of these groups. Based on local numbers, if we enroll fewer than 17% women and 12% minority veterans, we will increase our targeted recruitment of these groups.

For clinic referrals, participating therapists will provide information about the study to veterans in their routine clinical practice settings who have clinically significant PTSD symptoms, as in previous work by the study team [41, 42]. Interested veterans will be contacted by the local study team. The local study team will describe the study and complete screening items with the participant. If the veteran is interested and meets eligibility criteria, study staff will consent and enroll the patient, then administer the CAPS-5 and DIAMOND to determine final eligibility. If a patient does not meet the criteria for PTSD on the CAPS-5, the veteran will be disenrolled from the study. We will enroll participants if they (1) score ≥ 33 on the PCL-5 when prompted to respond to questions in reference to the index trauma, (2) meet criteria for PTSD on the CAPS-5, (3) meet other inclusion/exclusion criteria, and (4) provide informed consent agreeing to participate. To facilitate this process, we will use recruitment methods that were successful in our previous work, including posting flyers and providing promotional materials to therapists and other clinicians who may come into contact with potential participants. If patients are unable to engage in a warm handoff during their appointment, clinicians will complete a release of information form and provide to research staff to facilitate recruitment. Patients may also reach out to research staff with contact information provided to them by their clinician.

## Assignment of interventions: allocation

### Sequence generation {16a}

We will randomize participants to one of the 16 conditions on the day of their initial therapy visit. Randomization will be stratified by sex and site. This procedure will ensure that treatment groups are balanced for variables that may correlate highly with the primary outcome.

### Concealment mechanism {16b}

Stratified permuted block randomization assignments are performed using a computerized sequence that was generated by the study statistician prior to the study.

### Implementation {16c}

Potential participants from the three VAMCs (Ann Arbor, Cincinnati, and Salt Lake City) will complete initial screening activities with their local research study team. After screening, all subsequent enrollment activities will be completed by the Ann Arbor study team. Once a participant has been enrolled and attends their first therapy session, the Ann Arbor study team will randomize the participant to one of the 16 intervention conditions.

## Assignment of interventions: blinding

### Who will be blinded {17a}

Outcome assessors are blinded to condition assignment. Trial participants and therapists are aware of the condition to which the participant has been assigned.

### Procedure for unblinding if needed {17b}

Unblinding is not relevant since participants and therapists are aware of their assigned condition.

## Data collection and management

### Plans for assessment and collection of outcomes {18a}

We will conduct participant assessments at four time points: baseline, post-treatment, 3-month follow-up, and 6-month follow-up. We will pay participants for their time (via gift card) at the following rates: $30 for baseline, $30 for the post-treatment assessment, $40 for the 3-month assessment, and $50 for the 6-month assessment. Surveys will be conducted through Qualtrics FedRAMP electronic survey software. Patients will be also given the option to complete the measures over the telephone, via another VA-approved platform such as va.zoom.gov, via paper and pencil in person, or via mail as alternatives to Qualtrics if they do not have access to telecommunications. We will use VA-approved communication strategies such as a VA Outlook account using Azure Rights Management (Azure RMS) to email participants appointment scheduling and reminders.

#### Variables and measures

Table 2 displays all measures. The assessment battery will take ~ 70 min to complete. The primary outcome is PTSD symptoms across the 6 months of follow-up as measured by the CAPS-5 score.

#### Screening measure

Trauma history will be assessed with the *Trauma History Screen (THS)*, a 14-item self-report measure that inquires about exposure to 14 “high magnitude stressors” (including combat) [45]. The *PCL-5* is a 20-item self-report measure of PTSD symptoms as defined by the DSM-5, with strong internal consistency, test–retest reliability, and convergent and discriminant validity [38, 46]. We will prompt participants to respond to PCL-5 items in reference to their index trauma. To be included in the study, participants must score ≥ 33, which indicates a probable diagnosis of PTSD [38].

#### Primary outcome measure

*The Clinician-Administered PTSD Scale for DSM-5 (CAPS-5)* [37] is a structured interview measure of PTSD severity with excellent psychometric properties [47]. As with the PCL-5, the CAPS-5 will be anchored to the index trauma. The CAPS-5 will be administered by independent evaluators via telephone or video visit.

#### Secondary outcome measures

We will assess self-report PTSD symptoms at baseline, post-treatment, 3-month, and 6-month follow-ups using the *PCL-5*. We will assess functioning using the *Brief Inventory of Psychosocial Functioning (B-IPF)*, a validated 7-item instrument that assesses PTSD-related functional impairment in the domains of romantic relationships, family relationships, work, friendships and socializing, parenting, education, and self-care [48]. We will assess depression with the *PHQ-9*, a 9-item measure of depression with excellent internal and test–retest reliability as well as construct and criterion validity [49]. Suicide risk will be assessed with the *Columbia Suicide Severity Rating Scale (C-SSRS)*, a validated suicide risk assessment tool that assesses the severity of suicidal ideation in the past month and suicidal behavior in the past 3 months [50]. Pain-related functional impairment will be assessed with the *Pain Catastrophizing Scale*, a 13-item measure with strong internal and test–retest reliability and convergent validity [51]. Functional disability will be assessed via the short form *WHO Disability Assessment Schedule (WHODAS 2.0)*, a 12-item measure with strong psychometric properties that provides a global disability score as well as six domain scores: cognition, mobility, self-care, getting along with others, participation in society, and life activities [52]. Patient satisfaction will be assessed with the *Client Satisfaction Questionnaire (CSQ-8)*, a validated 8-item measure of satisfaction with treatment [53]. *Mental health and medical services utilization*: We will collect mental health (outpatient, inpatient, and pharmacy) and medical (primary care, emergency department, and inpatient) service utilization data for all VA services received in the 6 months prior to study enrollment and during the month period of study participation. We will assess receipt of trauma-focused treatment (e.g., CPT; Prolonged Exposure) during the follow-up period. This data will be collected by chart review.

#### Mediators

The brief *Posttraumatic Cognitions Inventory (PTCI-9)* is a 9-item measure of negative and dysfunctional cognitions that develop after traumatic events [54]. The PTCI-9 has strong internal and test–retest reliability [55]. Our team has demonstrated that changes in PTCI mediate the effects of CPT [29]. *Treatment Engagement/Adherence*: We will calculate engagement as percent of sessions attended and percent of homework assignments completed (assessed via a therapist-rated worksheet used in our previous work), which we have shown mediate the effects of CPT [56, 57]. Therapists will track participant homework completion at each therapy session using previously developed checklists.

#### Moderators

##### Demographics

We will collect information on sex, age, race, ethnicity, income, and other demographic characteristics from the *PhenX Toolkit Mental Health Research Core: Tier 1* [58]; combat era; comorbidities; service-connected disability status, and psychiatric medications. We will assess psychiatric comorbidities with the Diagnostic Interview for Anxiety, Mood, And Obsessive–Compulsive And Related Neuropsychiatric Disorders (DIAMOND) [59]. The DIAMOND is a semi-structured diagnostic interview for assessing psychiatric disorders. We will use the DIAMOND to assess for the presence of psychotic symptoms, unmanaged bipolar disorder, and the need for substance detoxification.

### Plans to promote participant retention and complete follow-up {18b}

Participants will be given materials at the onset of therapy with their corresponding schedule of study events. The study team will reach out to participants 2 weeks prior to the follow-up target date to schedule the necessary study activities and continue outreach throughout the window until the follow-up activity is completed or the window for that timepoint closes. Outreach efforts will be repeated for each timepoint. Surveys are sent via Qualtrics on the target date for each timepoint (baseline, post-treatment, 3-month, and 6-month) with up to 10 reminders.

Participants may choose to withdraw at any time without penalty. We will use data collected up until the point of withdrawal. Patients may withdraw from the intervention and continue to complete all follow-up assessments. The therapist may remove a patient from the study if it is deemed no longer in the patient’s best interest to participate (i.e., increased suicide risk, substance use, or condition requiring a higher level of care). Should a participant choose to discontinue with the study entirely, they will be immediately disenrolled and no further contact will be made to complete follow-up activities. Reasons for discontinuation or disenrollment will be recorded in our study database and our CONSORT diagram.

If individuals are withdrawn from care or elect to withdraw from the study interventions, we will encourage them to reach out to their local mental health treatment coordinator to determine the best treatment options for them.

### Data management {19}

#### Data management and cleaning

We will use Qualtrics to collect and manage study data. We will conduct data cleaning throughout the data collection period to ensure the appropriate production of a final dataset for analysis. We will use the VA Corporate Data Warehouse as the data source for mental health and medical service utilization data. We will use SAS (Statistical Analysis System) to examine and prepare data for analysis.

Data will be entered independently by trained data entry staff, and discrepancies will be corrected by a supervisor, based on source documents. Data will be analyzed using SAS software. Data quality will be monitored throughout the study by random inspection of the completed forms by the study coordinator, and any problems detected will be discussed with the PD/PI. Every effort will be made to ensure that we have accurate and complete data for all measures. The study will have both drop-outs and those lost to follow-up. We will prepare a CONSORT flow diagram to describe the disposition of veterans at each stage of the research. We will also collect the reason for missing data such as “patient refusal” or “scheduling complication” to have a better understanding of the mechanisms of missingness. We will check for patterns of missingness, compare the rate of missingness at each follow-up time and dropouts between groups, and compare reasons for dropouts between groups. We will also check if dropout depends on covariates and will include those covariates in the modeling procedures.

### Confidentiality {27}

All investigators and research staff have met VA training requirements for handling protected health information as defined by the Health Insurance Portability and Accountability Act (HIPAA), data security, and privacy. All data storage and handling will follow defined protocols at the VA Ann Arbor Healthcare System Center for Clinical Management Research. Throughout the study, IRB and HIPAA guidelines will be followed to ensure the privacy and integrity of the information we collect. Any breach will be immediately reported to the PD/PI and the IRB.

All study datasets will use confidential case identifiers. Data will be confidential but not anonymous, since personal identifiers are needed to link individual data across data sources. For this purpose, an electronic crosswalk file will be stored in a secure, access-limited folder on the Center’s server. All VA-generated electronic study data including participant identifiers such as patient names, phone numbers, physical mailing addresses, and health data will be securely maintained on a VA-restricted server in an access-limited folder, with access given only to specified project staff. All research findings will be presented in aggregate only.

### Plans for collection, laboratory evaluation, and storage of biological specimens for genetic or molecular analysis in this trial/future use {33}

No biological specimens for genetic or molecular analysis will be collected or stored for this study.

## Statistical methods

### Statistical methods for primary and secondary outcomes {20a}

#### Analytic strategy

To test the effectiveness of each CPT component, we will aggregate outcomes across conditions that contain that component. The primary analytic cohort will be intent-to-treat; we will analyze the impact of the assigned sessions, rather than the impact of sessions actually received.

Specific Aim 1: Using a highly efficient experimental design, identify which of five CPT components contribute meaningfully to reduction in PTSD symptoms (Primary Outcome)

We will test the effectiveness of each component and each two-way interaction between components, as measured by PTSD symptom reduction on the Clinician-administered PTSD scale for DSM-5 (CAPS-5) across 6 months of follow-up.

*Data Analytic Plan for Aim 1*: The analysis of the Primary Aim will test the main effects and two-way interactions between the five CPT components on change (decrease) in PTSD symptoms from baseline to month 6. Secondary outcomes include functioning, depression, patient satisfaction, and service utilization. There are a total of four measurement occasions for this analysis: PTSD symptoms measured at baseline (time = 0), post-treatment/6 weeks (time = 1.5), 3-month (time = 3), and 6-month (time = 6) post-randomization. Linear mixed models (LMMs) using SAS PROC MIXED will be used to analyze the longitudinal data. LMMs use all available outcome data, allowing subjects to have an unequal number of observations, and accommodating missingness when the response is missing at random [60]. The analysis will fit an LMM with fixed effects for the intercept, time, and interactions for time with each of the effect-coded components (On = 1 vs. Off =  − 1; see [32]) and with each two-way interaction between components. The LMM will also include random effects for the intercept and time (to account for within-person correlation) and will adjust for sex, age, site, and delivery modality (in-person versus telehealth). Model diagnostics will be used to determine the suitability of more parsimonious (e.g., autoregressive) correlation structures and nonlinear effects for time. From the fitted LMMs, the main effects and interactions between components will be examined by testing if the coefficients for their interactions with time are different from zero. For example, consider the coefficient for the interaction term between Challenging Beliefs and time. The value of six times this coefficient is an estimate of the mean difference in PTSD symptoms from baseline to 6 months between those who were offered vs. those not offered the Challenging Beliefs component. A component will be considered effective if its presence produces a statistically significant main effect or synergistic two-way interaction of Cohen’s *d* ≥ 0.25 (considered small to moderate) [61] and “possibly effective” if its main effect is significant but between 0.15 and 0.25. Specifically, following decision-making guidelines from Collins, [39] a component will be considered for inclusion in the optimized intervention if its main effect is statistically significant (*p* < 0.05) or has a magnitude ≥ 0.25 in terms of Cohen’s *d*. Components that do not meet the main effect criteria may be considered for inclusion if they are involved in a synergistic interaction of Cohen’s *d* ≥ 0.25. Conversely, a component meeting the main effect criteria may be considered for exclusion if it is involved in an antagonistic interaction of Cohen’s *d* ≥ 0.25. Using this paradigm, we will identify components that will comprise the optimized intervention.

Specific Aim 2: Identify mediators of component effectiveness

We hypothesize that effects will be mediated by engagement/adherence and change in posttraumatic cognitions.

*Data Analytic Plan for Aim 2*: We will test whether changes in the primary outcome of PTSD symptoms are mediated through engagement/adherence and changes in posttraumatic cognitions (PTCI-9). We will first evaluate adherence to treatment by reporting the percentage of sessions attended, percentage of homework completed, and changes in PTCI-9 scores at each post-randomization study time. Mediation analyses will be performed using structural equation models with intervention component main effects and interaction effects as independent variables, engagement/adherence or posttraumatic cognitions as the mediator, and CAPS-5 change as the outcome. The structural equation model will be fit using maximum likelihood estimation in the software program AMOS. In order to establish temporal ordering, we will examine whether, for example, PTCI scores at post-treatment and 3-month post-randomization mediate the effect of intervention components on 3- and 6-month CAPS-5 scores. We will first determine whether the component is associated with the hypothesized mediator over the first 3 months of follow-up and check for a direct effect of the mediator at post-treatment and 3 months on PTSD symptoms as measured at 3- and 6-month follow-ups. We will estimate the indirect effect of the intervention components on PTSD symptoms through the mediators and compute bias-corrected confidence intervals using 2000 bootstrapped samples [62, 63].

### Interim analyses {21b}

No interim analyses will be conducted.

### Methods for additional analyses (e.g., subgroup analyses) {20b}

#### Exploratory Aim 1: Identify moderators of component effectiveness

We will test whether intervention components are differentially effective by participant characteristics, including sex, age, and initial severity.

*Data Analytic Plan for Exploratory Aim 1*: We will explore whether intervention effects are moderated by sex, age, race/ethnicity, PTSD severity, and substance use. The model for this analysis will be identical to Aim 1 with the following exceptions: (1) interactions between components will not be included, unless they were significant in the primary aim; and (2) component-by-moderator interaction terms, as well as a component-by-moderator-by-time interaction terms will be added.

### Methods in analysis to handle protocol non-adherence and any statistical methods to handle missing data {20c}

#### Missing data for quantitative analyses

We will prepare a CONSORT diagram [64] to describe disposition of participants at each stage of the research. We will check for patterns of missingness, compare rates of missingness and dropouts between groups at each follow-up time point, and compare reasons for dropout between groups. We will also test whether dropout depends on baseline covariates and will include those covariates in the modeling procedures. Our analytic approach for the clinical outcomes of interest will be longitudinal data analysis via linear mixed models (LMMs). A notable strength of using LMMs is that this approach allows for the use of data from all participants (including those with only baseline measures) and provides unbiased parameter estimates under the missing-at-random assumption [60]. Although we cannot directly test the missing-at-random assumption, if missingness is greater than 10%, we will also conduct sensitivity analysis with multiple imputation procedures implemented with chained equations to determine whether results are consistent under different missingness assumptions such as missingness depending on baseline symptom severity [65].

### Plans to give access to the full protocol, participant-level data, and statistical code {31c}

A de-identified, anonymized dataset will be created and shared as appropriate. No audio recordings will be shared.

## Oversight and monitoring

### Composition of the coordinating center and trial steering committee {5d}

Dr. Sripada (PD/PI) will be responsible for ensuring the standardization of procedures among staff and investigators. The Project Manager will aid in the oversight of project personnel and the organization of project meetings. Communication among staff and investigators will be facilitated by a combination of phone and video meetings organized by the Research Coordinator.

### Composition of the data monitoring committee, its role and reporting structure {21a}

This project will be overseen by the VA HSR&D Data and Safety Monitoring Board. This Board provides guidelines on plans for monitoring safety of participants and the accuracy and integrity of the data, and reviews participant recruitment and enrollment. One task of this board will be to review any adverse events and early study results to make decisions about continuation. We will comply with all Board guidelines and requirements.

The members of the DSMB will have no direct involvement with the study or intervention. Should new information become available during the course of this research, which may indicate that the risks of harm have increased significantly, the investigators will inform participants so they may reconsider their willingness to participate.

### Adverse event reporting and harms {22}

The timing of the reporting of any adverse events to the VA Central IRB and DSMB by Dr. Sripada will be dependent on the severity of the event, and whether such adverse events were expected (i.e., included in the informed consent). Any SAE related to study intervention will be reported to the Central IRB and DSMB according to their reporting guidelines. A Serious Adverse Event (SAE) is any adverse experience occurring during the study that (a) results in death, (b) is life-threatening (e.g., imminent suicide risk, homicidality), (c) results in hospitalization or prolongation of hospitalization, or (d) results in persistent disability. 

The PD/PI will notify the VA Research Central Office project officer of any study modifications or suspension imposed by the IRB and/or DSMB in response to SAEs. Finally, if considered related to the trial, unanticipated problems involving risks to subjects or others will be reported to both the DSMB, the Central IRB, and then to Institution Officials who will promptly inform the VA Research Central Office and the Office for Human Research Protections (OHRP).

#### Risk assessment

Risk assessment is incorporated into the study at multiple timepoints. At initial screening, suicide risk is assessed by trained staff via the Columbia Suicide Severity Rating Scale (C-SSRS), a validated suicide risk assessment tool that assesses the presence of suicidal ideation in the past month and suicidal behaviors in the past 3 months. At baseline assessments, this information is collected again. Once patients connect with their assigned clinician, suicide risk is assessed during each therapy session. If a participant endorses suicidal ideation on the C-SSRS, we will ask additional questions regarding plan and intent. Depending on the participant’s responses, and in consultation with PD/PI Sripada, the study team member or CPT therapist will determine the appropriate disposition (e.g., create a safety plan; request wellness check; activate emergency medical services; escort the participant to the Emergency Department).

### Frequency and plans for auditing trial conduct {23}

Quality control and reliability of screening, baseline, and follow-up assessments will be monitored by Dr. Sripada throughout the trial via regular meetings and observation of the research staff conducting standardized assessments and throughout the study via regular meetings. Dr. Sripada will monitor the quality of the data files via the supervision of the data manager. The study team will meet regularly (at least bi-weekly) during the study period. Bi-weekly meetings of the research staff of this study will include a review of accrual, consenting procedures, protocol adherence, adverse events, and quality control of all data obtained from the study in the previous week. The PD/PI and study team will work to address any problems/events to reduce the likelihood of their recurrence and keep the risk to participants as low as possible. These team meetings will also ensure that all relevant IRB policies and study procedures are being followed. We will monitor site recruitment at least once a month and add additional sites if needed.

### Plans for communicating important protocol amendments to relevant parties (e.g., trial participants, ethical committees) {25}

Dr. Sripada will be responsible for submitting protocol amendments to the Central IRB. Protocol modifications are communicated verbally and in writing to study staff.

All research clinicians, study staff, and data analysts convene at biweekly all-team meetings to update on team-level issues, express needed changes, and decide on alterations. On a weekly basis, therapist consultation meetings allow therapists and staff members to meet and discuss patient progress, problems, solutions, and any needed protocol modifications, including analysis, results, and inclusion/exclusion criteria. Finally, bi-weekly research assistant meetings are held to address weekly and monthly recruitment rates, evaluate training needs, and facilitate inter-team communication on site-related variances. At VAMC sites, site investigators also hold weekly meetings with research assistants to evaluate recruitment progress, screening, and scheduling; and provide real-time answers to pressing questions.

### Dissemination plans {31a}

The study team comprises several leaders in the field of VA CPT delivery, and they will serve as champions to support uptake of study results. Our operations partner, the National Center for PTSD, will “own” the study results and disseminate the refined treatment if proven effective. Since VA PTSD education is coordinated by the National Center for PTSD, we will keep National Center leaders apprised of our findings and incorporate findings into regular National Center outreach efforts. Co-I Wiltsey-Stirman leads the Implementation Lab at the Dissemination and Training Division at the National Center for PTSD, which incubates and pilots VA psychotherapy rollouts. She also has access to a range of different tools to disseminate findings and practices, including the National PTSD Mentor Program, National Center for PTSD website, newsletters, and other mechanisms (see Letter of Support). In addition, Co-I Chard, as the original developer of CPT and the VA CPT Implementation Director, can disseminate results through the VA CPT program.

## Discussion

At the completion of the project, we expect to have determined which components of CPT produce the greatest reduction in PTSD symptoms. These components will be combined, along with the initial and final sessions, to produce an empirically-derived, brief version of CPT, which will be tested in a subsequent RCT. Providing the field with a brief, empirically-based version of CPT will improve care for veterans who desire a shorter course of PTSD treatment and can potentially be used to expand access to CPT to settings where only brief treatments are feasible.

### Potential problems and alternative strategies

One potential consideration is challenges with recruitment. To proactively prevent this problem from occurring, we carefully selected sites with multiple CPT providers, high numbers of eligible veterans, and a successful track record of conducting clinical trials with CPT. Furthermore, we have identified two additional sites that are interested and able to participate in this study. If we drop below our recruitment target for two consecutive months, we could expand recruitment to these two sites. We could also query the electronic health record at recruitment sites for veterans with new PTSD diagnoses or positive PTSD screens and proactively reach out to these veterans to assess their interest in the study. Thus, we have a number of ways to address any potential recruitment challenges.

Another potential problem is maintaining therapist fidelity. In order to test the effectiveness of individual CPT components, the different experimental conditions will entail the delivery of different combinations of CPT components, and it is imperative that therapists only deliver the CPT components that are assigned for a given condition. Standard psychotherapy trials monitor 20% of session recordings, but to ensure fidelity in this factorial trial, we will increase the proportion of monitored sessions to 50%. We will monitor these session audio recordings on a rolling basis and notify the therapist and the trainer if the fidelity of a session falls below 80%. Based on the strength of the study team’s fidelity monitoring procedures, [42] we expect this approach to lead to a mean of 4 (out of 7) for competence and 90% adherence. Nevertheless, it is possible that fidelity might fall below acceptable rates for a given therapist. In that unlikely event, we would provide additional training and consultation to the therapist, or, if this is unsuccessful, remove the therapist from the study.

It is possible that veterans will seek additional trauma-focused treatment (e.g., standard CPT or Prolonged Exposure therapy) during the 6-month follow-up period. However, given the limited number of CPT sessions received in general clinical practice, we believe this occurrence will be infrequent. Nevertheless, our intent-to-treat analysis will data from all study timepoints to test effects and to investigate non-linear effects for time (e.g., a particular component may have a strong effect during the first 6 weeks, which attenuates by month 6 due to possible exposure to other treatments between week 6 to month 6). Although the primary outcome is a change in PTSD symptoms by Month 6, we will also investigate possible trends over time. In addition, we will conduct post hoc sensitivity analyses with those who did and did not receive additional trauma-focused treatment during the 6-month follow-up period.

It is also possible that the CPT components tested in the factorial experiment will not produce differential effects on PTSD symptoms. We hypothesize that there will be differential effects based on previous psychotherapy research using factorial designs [66] and previous work by study team members [57]. However, if there are no components that exhibit clear superiority in terms of symptom reduction, we will select the components that are associated with the best adherence/lowest dropout. Although we are confident about our selected methods, having these strong alternatives bolsters our chance for success, should challenges arise.

One alternative strategy we considered was having the intervention delivered by a research therapist instead of existing providers. However, Collins recommends conducting factorial experiments in real-world conditions [31]. Furthermore, the use of existing providers will enhance the generalizability of our findings.

### Future directions

At the conclusion of this study, we will have developed a brief version of CPT. The brief version will be tested against full-length CPT in a full-scale non-inferiority trial. Pending demonstration of effectiveness, the brief treatment can be disseminated through the VA CPT training program, which is led by Co-I Chard. Disseminating a brief, effective version of CPT will help achieve the VA’s goal to increase the engagement and retention of veterans in evidence-based therapies for PTSD and will have a positive impact on the health and wellbeing of veterans with PTSD.

### Trial status

Protocol Version 4, 05/02/2023. Recruitment began 07/01/2022 and is expected to be completed by 06/30/2025.

### Supplementary Information

**Additional file 1.** Key summary information about this study.

**Additional file 2.**

## Data Availability

N/A.

## Abbreviations

B-IPF: Brief Inventory of Psychosocial Functioning
CAPS-5: Clinician-Administered PTSD Scale for DSM-5
CONSORT: Consolidated Standards of Reporting Trials
CPT: Cognitive processing therapy
CSQ-8: Client Satisfaction Questionnaire
C-SSRS: Columbia Suicide Severity Rating Scale
DOD: Department of Defense
DSM-5: Diagnostic and Statistical Manual of Mental Disorders, 5^th^ Edition
DSMB: Data Safety and Monitoring Board
EBP: Evidence-based psychotherapy
FedRAMP: Federal Risk and Authorization Management Program
HIPAA: Health Insurance Portability and Accountability Act
HSR&D: Health Services Research & Development
IRB: Institutional Review Board
LMMs: Linear mixed models
MOST: Multiphase optimization strategy
OHRP: Office for Human Research Protections
PCL-5: PTSD Checklist for DSM-5
PCTs: PTSD Clinical Teams
PD: Project Director
PHQ-9: Patient Health Questionnaire-9
PI: Principal Investigator
PII/PHI: Protected Identifiable Information/Protected Health Information
PTCI: Posttraumatic Cognitions Inventory
PTSD: Posttraumatic stress disorder
RA: Research assistant
RCT: Randomized control trial
RMS: Rights Management System
SAE: Serious adverse event
SAS: Statistical Analysis System
THS: Trauma History Screen
URL: Uniform Resource Locator
US: United States
VA: Veterans Affairs
VAMC: Veterans Affairs Medical Center
