# Supplementary material for: Using the multiphase optimization strategy to adapt cognitive processing therapy (CPT MOST): study protocol for a randomized controlled factorial experiment
Source: Trials. 2023 Oct 19;24:676. doi: 10.1186/s13063-023-07669-3 (PMC10588087; doi:10.1186/s13063-023-07669-3)
Supplement: Supplementary file 1 — Additional file 1. Key summary information about this study. [file 13063_2023_7669_MOESM1_ESM.docx]

**KEY SUMMARY INFORMATION ABOUT THIS STUDY**

You are being invited to take part in a research study that is being funded by VA HSR&D. Before you decide to take part, it is important for you to know why the research is being done and what it will involve. This includes any potential risks to you, as well as any potential benefits you might receive. Taking part in this study is completely voluntary.

**what is the STUDY ABOUT AND HOW LONG WILL IT LAST?**

By conducting this study, we hope to adapt, test, and implement a brief, evidence-based treatment for Veterans with PTSD. Specifically, we hope to identify the most effective intervention components of Cognitive Processing Therapy (CPT) so that CPT can be deliverable in a shorter timeframe, thus improving efficiency, reducing drop-out related to poor treatment response, and ensuring that Veterans receive the most beneficial components of treatment, which will significantly improve their quality of life. We will be enrolling 270 Veterans from multiple VA’s into this study for approximately 3.5 years, however, your participation will last for approximately 6 months and will include attending between 4-8 sessions of weekly CPT therapy and completing survey questionnaires/interviews when you enroll in the study and again 6-weeks, 3-months, and 6-months later.

**what are key reasons you might choose to volunteer for this study?**

##### The Department of Veterans Affairs (VA) recommends trauma-focused talk therapy as the best treatment option for PTSD

##### You will receive a talk therapy designed to improve you PTSD symptoms

- The talk therapy offered as part of this study is routinely used, known to be safe, and is a gold standard treatment recommended by the VA for treating PTSD
- This study is NOT testing experimental therapies, rather, we are testing how to deliver an existing therapy in a shorter time frame
- You will be helping to advance the scientific community’s understanding of how to improve access to evidence-based PTSD treatment

For a complete description of benefits, refer to the Detailed Information section of this form.

**what are key reasons you might choose not to volunteer for this study?**

- There is no guarantee you will personally benefit from taking part in this study
- Some survey questions may bother you
- There is a risk that someone not on the study team may see the information collected from you
- You may prefer to use only medication (and not therapy) to treat your PTSD
- You may find therapy to be distressing
- You may already be engaged in or prefer another trauma-focused treatment

*For a complete description of alternate treatment/procedures, refer to the Detailed Information section of this consent.*

**DO YOU HAVE TO TAKE PART IN THE STUDY?**

If you decide to take part in the study, it should be because you really want to volunteer. You will not lose any services, benefits or rights you would normally have if you choose not to volunteer.

**what if you have questions, suggestions or concerns?**

The person in charge of the study is Rebecca Sripada, PhD at the Ann Arbor VA. If you have questions, suggestions, or concerns regarding this study or you want to withdraw from the study his/her contact information is:

Rebecca Sripada, PhD

VA Center for Clinical Management Research

VA Ann Arbor Healthcare System

NCRC Bldg 16

2800 Plymouth Road

Ann Arbor, MI, 48105

Email: rebecca.sripada@va.gov

**DETAILED INFORMATION ABOUT THE STUDY**

**WHAT IS THE PURPOSE OF THIS STUDY?**

By conducting this research, we hope to develop a briefer model of CPT, which would likely improve clinical care for Veterans with PTSD. By conducting this research project, we hope to:

- Identify which of the CPT components contribute meaningfully to decreases in PTSD symptoms
- Assess the impact of demographic characteristics, treatment engagement, and other factors on the brief model of CPT

**HOW LONG WILL I BE IN THE STUDY?**

The overall length of this study is three and half years. Your participation in the study would last for about six months.

**WHAT WILL HAPPEN IF I TAKE PART IN THE STUDY?**

If you agree to participate, you will be randomized by a computer (like the flip of a coin) to determine how many sessions of CPT you will participate in. You will receive between four and eight sessions of CPT. All therapy sessions will be delivered by regular members of your VA health care team. We will audio record your therapy sessions. The research team will review these recordings to make sure your therapist is providing the therapy as intended. We will encourage you and your therapist to start the treatment as soon as you are enrolled. These sessions may occur in person or via video depending upon your facility’s policies as well as both you and your provider’s preferences.

- Symptom Interview: Regardless of how many sessions you get randomized to or how many sessions you complete, you will also be asked to complete 4 interviews asking about your PTSD symptoms. These interviews will be administered by staff via phone or Video Connect. We will send you a gift card for completing each interview.
  - The first interview will occur shortly after your enrollment into the study. It is possible that after completing this first interview, the provider will determine that you do not meet criteria for PTSD at this time. If this is the case, you will not participate in any therapy sessions and your participation in this study will be complete. If you do meet criteria, the next three interviews will be conducted at 6-weeks, 3-months, and 6-months after you are randomized. These interviews will take approximately 45 minutes to complete.
- Surveys: Regardless of how many sessions you get randomized to or how many sessions you complete, you will be asked to complete 4 surveys. These surveys will be administered by staff from your local facility as well as staff from the Ann Arbor VA facility. You can complete these surveys on the web, over the phone, or on paper via mail. The surveys will ask questions about your overall health and mental health, attitudes about treatment, social support, and substance use. You can skip any questions you do not wish to answer. We will send you a gift card for completing each survey.
  - The first survey will occur after you are enrolled and before you begin your therapy sessions. The next three surveys will be conducted 6-weeks, 3-months, and 6-months after you are randomized. These surveys will take approximately 30 minutes to complete.
- Review of Medical Records: The research team will collect information from your VA medical record. We will not need to look at all your records. Instead, we will use a computer to find information about your use of health care services, including:
  - Outpatient mental health visits
  - Inpatient mental health visits
  - Medications
  - Outpatient medical visits
  - Emergency room visits
  - Inpatient medical visits

We will collect this information for the six months prior to your enrollment into the study and for the 6 month duration that you are in the study.

**WHAT IS EXPECTED OF ME IF I TAKE PART IN THIS STUDY?**

- Keep your therapy appointments. If you know you will need to miss an appointment, please call your clinic/therapist ahead of time to reschedule as soon as possible.
- Keep your study appointments (surveys and interviews). If you know you will need to miss an appointment, please call the study team ahead of time to reschedule as soon as possible.
- Complete all study surveys
- Allow us to collect information from your medical record
- Ask questions as you think of them
- While participating in this study, do not take part in another study without permission from one of the investigators. Taking part in another study may invalidate the results of both studies.
- We are asking each study participant to give us the names and telephone numbers of at least two people (relatives, friends, or neighbors) whom we may call if we have difficulties contacting the participant during the study. The study team will *only* call these people if they have tried other ways of contacting you and if you have not asked to end your study involvement. You can leave the study whenever you desire, and should you choose not to remain in this study, you will not be expected to continue to meet nor be contacted further by research staff.

**WHAT POSSIBLE RISKS OR DISCOMFORTS MIGHT I HAVE IF I TAKE PART IN THIS STUDY?**

Any procedure has possible risks and discomforts. The procedures in this study may cause all, some, or none of the risks or side effects listed. Rare, unknown, or unexpected risks also may occur.

Therapy: Trauma-focused talk therapy has been proven to be effective and safe. It is recommended by the *VA/DoD Clinical Practice Guidelines for PTSD*. It is used in routine care. However, there are known risks and discomforts.

- Talking about trauma with a therapist can be uncomfortable. Your therapist will talk with you about this, and help you cope with any distressing thoughts or disturbing memories.

Surveys/Interview: Answering survey and interview questions takes time and you may find this inconvenient. We will do our best to schedule the survey and interview at a time that fits your schedule. You are also allowed to take as many breaks as necessary during the survey and interviews. The survey and interview also ask some uncomfortable questions about sensitive issues.

- Uncomfortable Questions: Some of the survey and interview questions may make you feel uncomfortable. An example of such a question is “In the past month, how much were you bothered by having strong negative feelings such as fear, horror, anger, guilt, or shame?” You can skip any question you do not feel like answering.
- Loss of Privacy of Sensitive Information: Some of the survey and interview questions ask about sensitive issues like using alcohol or drugs. A risk of participating in this study is possible loss of privacy of this sensitive information. This is very unlikely, but we cannot absolutely guarantee that it won’t happen. We will protect your information in the following ways:
  - We will identify you only by a study ID on all files, so that no one can link your data to you directly.
  - All your answers to the survey questions will remain strictly confidential. Survey and interview data will be stored on a secure server at the Ann Arbor VA behind the VA firewall. Only study staff will have access to the files.
  - We will not share your information or survey/interview answers with anyone outside of the study team without your written permission. However, if we feel you are in danger of harming yourself, we are required to get help for you.
  - When the results of this study are reported, your data will be combined with other patients. You will not be identified in any way. For example, we will report the percent of patients saying “not at all” to a survey question about feeling depressed.

There is always a chance that any procedure can harm you. The procedures in this study are no different. In addition to the risks described above, you may experience a previously unknown risk or side effect.

Risks of the usual care you receive are not risks of this study. Those risks are not included in this consent form. You should talk with your health care providers if you have any questions about the risks of usual care.

**WHAT ARE THE POSSIBLE BENEFITS OF THIS STUDY?**

If you participate in this study you will receive a therapeutic intervention designed to improve your PTSD symptoms, however, you may not receive any direct benefits for participating in this study. However, if the this study is demonstrated to be effective, the findings may be used to implement a briefer model of CPT in the VA, which could improve efficiency, reduce drop-out related to poor treatment response, and ensure that Veterans receive the most beneficial components of treatment, which will significantly improve their quality of life.

**WHAT OTHER CHOICES DO I HAVE IF I DO NOT WANT TO JOIN THIS STUDY?**

If you do not take part in this study, you have other options.

- You can stick with the treatment you are currently receiving
- You can ask your provider for a referral to a clinic which offers a variety of trauma-focused talk therapies known to be effective for PTSD
- You can speak to your provider about starting a medication treatment for your PTSD

**HOW WILL MY PRIVATE INFORMATION BE PROTECTED?**

Taking part in this study will involve collecting private information about you.  This information will also be shared with staff at the Ann Arbor VA, which is the coordinating center for this study.

This information will be protected in the following ways:

- Paper study records will be kept in a locked filing cabinet in a locked VA space
- Electronic study records and audio files will be stored in secure study folders on VA computer servers
- Only approved research staff will have access to the information, including that necessary to compensate you for your participation.
- If the results of this study are reported in medical journals or at meetings, you will not be identified by name or by any other means.
- If we learn that you intend to harm others, we must report that to the authorities. If you report that you intend to harm yourself, we will connect you with professionals trained in suicide prevention.

Your information collected as part of the research, even if information that identifies you is removed, will not be used or distributed for future research studies. By law, study records must be kept in a secure location for about six years after the study has ended, at which time they will be destroyed in accordance with VA policy.

While this study is being conducted, you will not have access to your research related health records. This will not affect your VA healthcare including your doctor's ability to see your records as part of your normal care and will not affect your right to have access to the research records after the study is completed.

A description of this clinical trial will be available on <http://www.ClinicalTrials.gov> as required by U.S. Law. This website will not include information that can identify you. At most, the website will include a summary of the results. You can search this website at any time.

**Health Information Portability and Accountability Act (HIPAA)**

There are rules to protect your private information. Federal and state laws and the federal medical Privacy Rule also protect your privacy. By signing this form, you provide your permission called your ‘authorization,’ for the use and disclosure of information protected by the Privacy Rule.

The research team working on the study will collect information about you. This includes things learned from the procedures described in this consent form. The study team may also collect other information including your name, address, date of birth, and information from your medical records such as HIV status, drug, alcohol or STI treatment, genetic test results or mental health treatment.

The research team may also need to disclose the information to others as part of the study progress. Others may include the following: Office of Human Research Protections (OHRP), the VA Office of Research Oversight (ORO), the Government Accountability Office (GAO; Sponsors; Contractors, Affiliates as appropriate), the VA Institutional Review Board, and the local VA medical facility Human Research Protections Program. These official organizations sometimes review studies to make sure they are being done safely and legally. If a review of this study takes place, your records may be examined. These reviewers will protect your privacy.

Your health information disclosed pursuant to this authorization may no longer be protected by Federal laws or regulations and may be subject to re-disclosure by the recipient.

While this study is being conducted you will not have access to your research related health records.

This will not affect your VA healthcare, including your doctor’s ability to see your records as part of your normal care and will not affect your right to have access to the research records after the study is completed.

You can revoke this authorization, in writing, at any time. To revoke your authorization, you must write to the Release of Information Office at this facility or you can ask a member of the research team to give you a form to revoke the authorization. Your request will be valid when the Release of Information Office receives it. If you revoke this authorization, you will not be able to continue to participate in the study. This will not affect your rights as a VA patient to treatment or benefit outside of the study.

If you revoke this authorization, **Rebecca Sripada, PhD** and her research team can continue to use information about you that was collected before receipt of the revocation. The research team will not collect information about you after you revoke the authorization.

Treatment, payment or enrollment/eligibility for benefits cannot be conditioned on you signing this authorization. This authorization will expire at the end of the research study unless revoked prior to that time.

**WHAT ARE THE COSTS TO ME IF I TAKE PART IN THIS STUDY?**

If you usually pay co-payments for VA care and medications, you will still pay these co-payments for VA talk therapy sessions you receive as part of this study.

**WILL I BE PAID FOR PARTICIPATING IN THIS STUDY?**

You will receive a gift card (i.e., Amazon, Walmart, Target, or similar) to compensate you for completing each of the following study activities:

- Enrollment survey ($15)/interview ($15) ($30 total)
- 6-week survey ($15)/interview ($15) (30 total)
- 3-month survey ($20)/interview ($20) ($40 total)
- 6-month survey ($25)/interview ($25) ($50 total)

Bonus per interview of $5 ($20 total)

At each time point a $5 bonus will be offered to participants that attend their originally scheduled clinician administered symptom interview appointment, as scheduled and without any reschedules, amounting to a maximum bonus of $20. Any incurred bonus payment will be delivered at the end of the 6-month follow up window*.* Only study staff will have access to your information, including information necessary to compensate you for your participation.

**WHAT WILL HAPPEN IF I AM INJURED BECAUSE OF MY BEING IN THE STUDY?**

If you are injured as a result of taking part in this study, the VA will provide necessary medical treatment at no cost to you unless the injury is due to non-compliance by a study participant with study procedures or if the research is conducted for VA under contract with an individual or non-VA institution.

Participants do not give up any legal rights or release the VA from any liability by signing this form.

If you believe you have been injured as a result of participating in this study, you can contact:

Rebecca Sripada any time

**DO I HAVE TO TAKE PART IN THE STUDY?**

It is up to you whether to take part in this study. You may decide not to be in the study at any time. If you choose not to take part or choose to stop taking part at any time, you will still receive the same health care and benefits from the VA you currently receive. Your decision will not change that in any way.

If you choose to take part in the study but then change your mind, Dr. Sripada and her research team can continue to use the information collected from you up to that point. The research team will not collect information about you after you let them know you no longer wish to take part in the study, though they may still collect information about you that is available from public records.

**RIGHT OF INVESTIGATOR TO TERMINATE MY PARTICIPATION**

The Research Team may withdraw you from the study without your consent form one or more of the following reasons:

- We believe that it is not in your best interest to stay in the study
- You become ineligible to participate
- You do not follow the instructions from the research team
- Your behavior toward the staff or other participants is disruptive or inappropriate
- The study is suspended or cancelled

**WHO DO I CONTACT ABOUT THIS STUDY IF I HAVE QUESTIONS?**

If you have any questions, complaints or concerns about the study you can contact the principal investigator:

Rebecca Sripada, PhD

VA Center for Clinical Management Research

VA Ann Arbor Healthcare System

NCRC Bldg 16

2800 Plymouth Road

Ann Arbor, MI, 48105

Email: rebecca.sripada@va.gov

You may also contact your local patient advocate.

If you have questions about your rights as a study participant, or you want to make sure this is a valid VA study, you may contact the VA Central Institutional Review Board (IRB). This is the Board that is responsible for overseeing the safety of human participants in this study. You may call the VA Central IRB toll free at 1-877-254-3130 if you have questions, complaints or concerns about the study or if you would like to obtain information or offer input.

**WILL I BE TOLD NEW INFORMATION ABOUT THIS STUDY?**

The researchers will tell you if they learn of important new information that may change your willingness to stay in the study. If new information is provided to you after you have joined the study, it is possible that you may be asked to sign a new consent form that includes the new information.

FUTURE USE OF DATA AND RE-CONTACT

The data file that contains your responses to survey and interview questions and review of your medical records but NOT any identifying information about you (e.g., name, address) may be shared with other researchers without additional permission from you. The stored data will not include any information that could identify you or link you to study participation. Researchers interested in accessing the data must submit a data request form. De-identified data will be provided after requesters sign a Letter of Agreement detailing the mechanisms by which the data will be kept secure and access restricted to their study team. The agreements will also state the recipient will not attempt to identify any individual and will not share the data with anyone outside of their research team.

**AGREEMENT TO PARTICIPATE IN THE RESEARCH STUDY**

The study coordinator, research assistant, or local site investigator has explained the research study to you. You have been told of the risks or discomforts and possible benefits of the study. You have been told of other choices of treatment available to you. You have been given the chance to ask questions and obtain answers.

By signing this document below, you voluntarily consent to participate in this study and authorize the use and disclosure of your health information in this study. You also confirm that you have read this consent, or it has been read to you. You will receive a copy of this consent after you sign it.

| **I agree to participate in this research study as has been explained in this document.** | | |
| --- | --- | --- |
| _________________________  Participant’s Name | ____________________________  Participant’s Signature | ___________  Date |
